# Diabetic and Metabolic Programming: Mechanisms Altering the Intrauterine Milieu

**DOI:** 10.5402/2012/975685

**Published:** 2012-11-20

**Authors:** Claudia Eberle, Christoph Ament

**Affiliations:** ^1^Medical Clinic and Policlinic IV, Ludwig Maximilian University of Munich, 80336 Munich, Germany; ^2^Division of Endocrinology, Diabetes and Clinical Nutrition, University Hospital of Zurich, Raemistrasse 100, CH-8091 Zurich, Switzerland; ^3^Institute for Automation and Systems Engineering, Ilmenau University of Technology, 98684 Ilmenau, Germany

## Abstract

A wealth of epidemiological, clinical, and experimental studies have been linked to poor intrauterine conditions as well as metabolic and associated cardiovascular changes postnatal. These are novel perspectives connecting the altered intrauterine milieu to a rising number of metabolic diseases, such as diabetes, obesity, and hypercholesterolemia as well as the Metabolic Syndrome (Met S). Moreover, metabolic associated atherosclerotic diseases are connected to perigestational maternal health. The “Thrifty Phenotype Hypothesis” introduced cross-generational links between poor conditions during gestation and metabolic as well as cardiovascular alterations postnatal. Still, mechanisms altering the intrauterine milieu causing metabolic and associated atherosclerotic diseases are currently poorly understood. This paper will give novel insights in fundamental concepts connected to specific molecular mechanisms “programming” diabetes and associated metabolic as well as cardiovascular diseases.

## 1. Introduction

Type 2 diabetes, obesity, and associated metabolic as well as cardiovascular diseases run an epidemic wave worldwide. Regarding to the World Health Organization (WHO) there are 347 millions people diagnosed with diabetes worldwide [1]. Furthermore, based on WHO fact sheets more than 1.4 billion adults were diagnosed overweight, in 2008, and of these more than 200 million men and approximately 300 million women were documented being obese [1]. Hence, over 40 million children under the age of 5 years were diagnosed overweight in the year 2010 [1]. Causes are seen in life style factors, such as high carbohydrate, respectively, high fat diets, and lack of exercise leading to obesity causing insulin resistance, type 2 diabetes and beta cell dysfunction, as well as associated metabolic and cardiovascular diseases. 

But, there is more and more evidence, that maternal peri-gestational genomic and environmental conditions may “imprint” metabolic and cardiovascular conditions in their offspring. Furthermore, novel insights underline important molecular and epigenetic changes in dysmetabolic pregnancies altering the intrauterine environment featuring evident programming concepts of metabolic and cardiovascular diseases in these offspring. Fundamental concepts of molecular and epigenetic mechanisms as well as key functions of the uteroplacental unit are starting to emerge quickly. Based on highlighted literature, especially Fernandez-Twinn et al. [2], this paper will give novel and future-directed insights in essential concepts linked to molecular mechanisms of diabetic and metabolic programming.

## 2. Epidemiological Facts 

**Figure 1:**
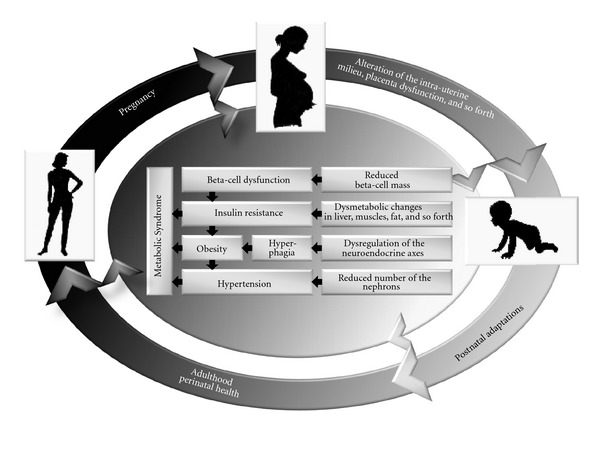
The “Thrifty Phenotype Hypothesis.” Modified from [2].

The term “programming” defines the perturbation at (a) critical period(s) of development causing permanent lifelong alteration(s) with irreversible consequences. An epidemiological key work connecting poor fetal growth and subsequent development of impaired glucose tolerance and non-insulin-dependent diabetes, as well as hypertension and cardiovascular disease, for example, was presented by Hales and Barker (“Fetal Origin Hypothesis”) [3, 4]. The authors could show that men in their 60s, who had documented lower weights at birth and throughout their first year of life, were more susceptible to develop impaired glucose tolerance, non-insulin-dependent diabetes and hypertension as well as cardiovascular disease [2–4]. These insights induced the “Thrifty Phenotype Hypothesis” connecting embryonic, respectively, fetal “malnutrition” to altered metabolic as well as associated conditions postnatal (Figure 1) [2]. Some of these modifications may have permanent lifelong consequences leading to metabolic, cardiovascular as well as associated diseases later on [2, 5]. Coming from the “Thrifty Phenotype Hypothesis,” Gluckman et al. reached out for the “Predictive Adaptive Responses Hypothesis,” which underlines that the embryo, respectively, fetus estimates and “predicts” the postnatal environment by “adapting” developmental processes already *in utero *[2, 6]. 

However, not having mapped the entire complexity of peri-gestational imprinting factors as well as vulnerable programming time windows within the *in utero* as well as *ex utero* development, yet, this altered *in utero* development may be altered again, postnatal, by nutritional and life style as well as environmental factors, for example, causing adverse metabolic and cardiovascular diseases later on [2]. To exemplify, the Dutch Hunger Winter respectively Dutch Famine Study showed increased numbers of adults diagnosed with impaired glucose tolerance and obesity, who were fetuses at the time of consequent alimentary rationing about the end of World War II [2, 7]. This study raised another important “programming” feature, emphasizing the importance of vulnerable “timing periods,” respectively, “developmental episodes” determining long-term consequences [2, 7]. It could be displayed that people who were fetuses in their particularly third trimesters during this period of famine had the worst metabolic outcome later in life as adults [2, 7]. 

Other important observations were documented in twin studies [2, 8–10]. By comparing twins with lower birth weights to twins with higher birth weights, it could be displayed that twins with lower birth weights were more susceptible to altered glucose tolerance, type 2 diabetes, and cardiovascular diseases, for example, see studies in [2, 8, 9]. Nowadays, clear related connections between low birth weight, but also high birth weight, and the susceptibility of postnatal metabolic, cardiovascular, and associated changes have been described [5, 11–13]. But since birth weight seems to be connected to metabolic and cardiovascular diseases in a “u-shaped” relationship lots of epidemiological, experimental, and clinical studies have focused on “programming” mechanisms within this transgenerational setting. Today, it seems presumably that especially maternal obesity and gestational diabetes as well as pregestational maternal diabetes are one of the strongest programming factors causing fetal malformations, altered birth weights as well as metabolic and cardiovascular diseases in their offspring postnatal. 

By following the “Fetal Insulin Hypothesis,” genetically determined alterations within the insulin signaling cascades, which may, for example, result in impaired glucose tolerance and insulin resistance, could be connected to altered fetal development as well as long-term metabolic changes [2]. Meanwhile, further studies support this hypothesis, for example, by linking mutations in the glucokinase gene (maturity onset diabetes of the young, MODY 2) to reduced birth weights [2]. Lindsay et al. [14] showed by investigating specific polymorphisms, which are associated with altered glucose tolerance and insulin resistance, that birth weights might be clearly influenced peri-gestational [14]. Studies, which compared genetic influences, for instance, polymorphisms or mutations, and environmental effects on the embryo, respectively, fetus, suggest that imprinting mechanisms and interaction effects caused by environmental factors might be strongly related to the outcome of metabolic and cardiovascular diseases later in life [2, 15]. But, still deeper insights analyzing the power and outcomes of different effects on both generations need to be analyzed more in detail. At least, these analyses provide evidences that impaired glucose tolerance, respectively, type 2 diabetes may have genetic aspects.

However, there is a broad spectrum of predominantly maternal influences, which are clearly linked to altered intrauterine environment and therefore associated with an altered metabolic and cardiovascular long-term outcome in the offspring. Representatives within this trans-generational metabolic programming are, for example, maternal pre-gestational diabetes as well as gestational diabetes, motherly obesity as well as raised maternal age, and maternal hypercholesterolemia as well as poor or inadequate nutrition, including macro- and micronutrient deficiency [2]. Further, maternal smoking, motherly stress factors, maternal hypertension, and pregnancy-related complications, such as (pre-) eclampsia, processes of systemic inflammation, and hypoxia, as well as combined dysmetabolic conditions, such as the Metabolic Syndrome, cause alterations in the intrauterine milieu [2]. This altered intrauterine environment is strongly associated with modified embryo-fetal development, which may be caused due to different molecular, respectively, epigenetic pathways *in utero* related to structural and functional alterations during development of the offspring. 

Given these facts, the embryo-fetal development in general, but also several organ functions in the offspring, such as the endocrine, immune, or hematopoetic system, may be modified due to altered *in utero* environment causing, for instance, short, as well as long-term metabolic and cardiovascular changes. An altered embryo-fetal development is often associated with modified pancreas mass, impaired insulin secretion, changed brain and neural development, altered adipose and skeletal muscle structure, including impaired insulin and cytokine signaling and embryo-fetal hypoxia as well as diversified kidneys and cardiovascular structures. 

Still, the mechanisms of developmental programming are poorly understood.

## 3. Experimental and Clinical Facts

**Figure 2:**
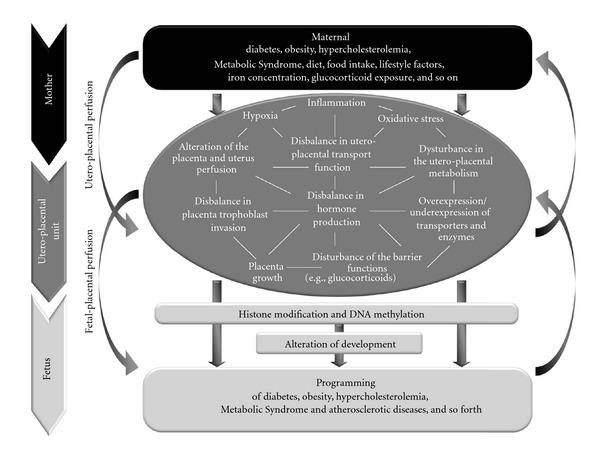
Mechanisms altering the intrauterine milieu causing diabetic, metabolic and cardiovascular programming.

Common risk factors linked to altered *in utero* milieu as well as long-term metabolic and cardiovascular consequences are, for instance, maternal peri-gestational diabetes, respectively, gestational diabetes, increasing maternal age, motherly obesity, and maternal macro- as well as micro-nutrient restrictions and shifting of nutrient contents, such as high fat or carbohydrate diets as well as low protein eating (Figure 2) [2–7, 16–20]. Furthermore, maternal hypoxia, iron restriction, endotoxemia, and glucocorticoid exposure as well as placental insufficiency are linked to alterations within the intrauterine milieu, poor or altered fetal growth, malformations and long-term metabolic as well as cardiovascular changes in the offspring [2–7, 14, 16–27]. These aspects are just representatives for numerous factors, which are mostly applied to poor developmental intrauterine environment and gestational, but also postnatal changes (Figure 2) [2–7, 16–28]. 

### 3.1. Focusing on Experimental Facts

*In vivo* models are widely used to analyze molecular, mechanistic, and phenotypic changes within the trans-generational metabolic and cardiovascular programming [2, 15–17, 20, 22–24, 28]. Especially, rodent models possess considerable advantages, such as a shorter gestational period as well as higher numbers of offspring, providing the possibility to analyze complex trans-generational mechanisms in both generations, but also, for example, the common accessible uteroplacental unit.

#### 3.1.1. Pregestational and Gestational Diabetes

Maternal pregestational diabetes respectively, gestational diabetes as well as maternal, obesity are considered to be one of the strongest imprinting factors in terms of metabolic programming [2, 20, 23, 24, 28]. Gestational diabetes ranks as one of the inducing factor for impaired glucose tolerance and type 2 diabetes in the offspring by altering the *in utero* milieu causing permanent defects in glucose homeostasis and type 2 diabetes in subsequent generations later in life (Figure 2) [2, 20]. Influences of maternal diabetes on their offspring can be modulated by streptozotocin (STZ). STZ, a chemical which destroys *β*-cells, is given by dose-dependent schemes leading, for example, either to weaker or stronger forms of (gestational) diabetes [2, 24]. Several studies displayed hyperinsulinemia, islets alterations, changes in insulin secretion, macrosomia, for example, in their offspring [2, 24]. In analogy to the epidemiological and experimental studies, gestational diabetes may lead to either low birth weight or high birth weight [2, 20, 24, 29]. 

#### 3.1.2. Preconceptional Obesity as well as Gestational Weight Gain

Maternal obesity before and when entering pregnancy is one of the crucial metabolic and cardiovascular imprinting factors in their offspring (Figure 2) [2, 13, 25, 26]. It has been described that maternal obesity causes altered insulin and leptin concentrations, which may cause endocrine modifications related to its synthesis, secretion, or action, leading to obesity in their offspring [2, 13, 23, 27]. Next to the maternal body weight per se experimental studies showed that maternal leptin concentrations may be linked to fetal key pathways [27, 30] causing metabolic changes in the next generation. However, targeting maternal obesity, Armitage et al. emphasizes the consequences of exposure to an energy rich diet during development [25]. Therefore, maternal overnutrition may act as an imprinting stimulus leading to metabolic changes in their offspring [25]. But also insulin and leptin concentrations may work as target programming hyperphagia, obesity, insulin resistance, and diabetes in the offspring. Having these concepts in mind, numerous studies analyzing courses of different gestational weight gain (GWG) and its outcomes on the offspring have been done [29, 31]. Still, the impact of GWG on both generations seems to be variable, but also associated with additional features. Further studies to gain deeper insights need to be done. 

#### 3.1.3. Pregestational and Gestational Hypercholesterolemia

However, it has been reported that altered maternal saturated fatty acid intake and hypercholesterolemia are harmful during the embryo-fetal development [32–36]. Palinski et al. displayed that maternal hypercholesterolemia could be linked to early postnatal metabolic changes, and connected to increased atherosclerotic plaque rates at early developmental stages in their offspring [32]. Therefore, more evidence is given that maternal nutrient patterns and metabolic constitutions alter molecular as well as phenotypic outcomes in offspring of dyslipidemic mothers. Today, maternal dietary habits mimicking the Western life style seem to be clearly linked to metabolic and cardiovascular diseases in their offspring.

#### 3.1.4. Maternal Macro- and Micronutrient Restriction

Not only maternal overnutrition but also maternal macro- as well as micronutrient restriction seems to play a crucial role of metabolic and cardiovascular programming. However, maternal food restriction up to 50% of *ad lib *during the last week of pregnancy causes an impairment of *β*-cell development in the offspring [2, 37]. Additionally, Garofano et al. stated that pancreatic “differentiation may be altered at fetal stages while proliferation may be affected after weaning” [37]. Therefore, by continuing the calorie restriction during the suckling period, the offspring possess a permanent reduction in *β*-cell mass and impaired glucose tolerance. In consequence, maternal food restriction up to 70% of *ad lib *resulted in hyperinsulinemia, hyperphagia, obesity and hypertension [2, 38]. 

A very well-studied *in vivo* model displays the maternal low protein model. For example, Snoeck et al. showed the linking of maternal low protein diet and lower birth weights in the offspring as well as decreased beta-cell proliferation and islet size in neonates or even altered adipocyte properties [39]. Focusing on the postnatal development of offspring which were *in utero* imprinted by maternal low protein intake, it could be reported that they experienced an age-dependent deficit of glucose tolerance causing impaired glucose tolerance, insulin resistance and diabetes by an age of 17 months at the latest [16]. Moreover, Ozanne and her colleagues, for example, could show that muscle insulin resistance in growth-restricted offspring is connected to an altered expression of Protein Kinase C zeta [40], by analyzing a low protein model. 

#### 3.1.5. Intrauterine Artery Ligations

There is rich evidence that placental insufficiency, for example, due to intrauterine unilateral or bilateral artery ligations lead to impaired nutrient perfusion through placenta and therefore to intrauterine growth retardations [2, 20, 41–43]. Unilateral as well as bilateral artery ligations are thought to cause placental insufficiency altering the metabolic outcome of the offspring. Boloker et al. described that offspring of uterine ligated dams showed reduced *β*-cell mass and became clearly diabetic already postnatal early on [20]. But, it is also discussed that consequences of artery ligations may affect the next generation even more. Boloker et al. displayed altered insulin secretions already at an age of approximately 5 weeks and diabetic conditions already at an age of 26 weeks in the next generation [20]. Furthermore, it is discussed that there may be a gender different outcome additionally.

#### 3.1.6. Maternal Hypoxia

Maternal hypoxia seems to have clearly impact on the metabolic as well as cardiovascular outcome in their offspring. However, maternal hypoxia may be linked to placental hypoxia causing altered fetal metabolic as well as phenotypic changes. But also cardiovascular diseases could be targeted by following chronic prenatal hypoxic conditions. Therefore, Li et al. respectively Peyronnet et al. showed that prenatal chronic hypoxia could be linked to low birth weights and significant increases in the susceptibility of adult cardiovascular diseases (Figure 2) [44, 45]. 

#### 3.1.7. Maternal Glucocorticoid Exposure

Maternal peri-gestational glucocorticoid exposure seems to play a crucial role imprinting phenotypic, and metabolic as well as cardiovascular changes in their offspring [46]. It has been discussed that maternal prenatal glucocorticoid exposure could be linked to reduced birth weights postnatal and hyperglycemic as well as hypertensive conditions in their offspring in their adulthood [14]. Lindsay et al. hypothesized that 11*β*HSD2 activity may be altered causing low birth weights and metabolic long-term consequences in the offspring [14].

### 3.2. Focusing on Clinical Facts

Underlining epidemiological and experimental studies, several clinical studies support different trans-generational programming concepts connecting these insights to long-term metabolic and cardiovascular diseases in the offspring. By focusing on clinical analyses, most phenotypic changes not only in mothers but also in their children have been highlighted. 

#### 3.2.1. Maternal Obesity

Maternal obesity could be identified as one of the fundamental metabolic and cardiovascular imprinting factors on their offspring. But to assess increased risk of metabolic and cardiovascular malprogramming early on birth weight studies targeting trans-generational aspects and mechanistic as well as phenotypic changes have been investigated. Having in mind that lower and higher birth weights are connected in a “u-shaped” way [11] to an increased risk of metabolic risk, especially type 2 diabetes, additional focus on changes in fat proportions, body mass index (BMI), appetite control and mechanisms of hypothalamic pituitary axis, insulin secretion and sensing as well as vascular responsiveness are discussed to imprint offspring of obese mothers [13]. Since there is a trend that mothers seem to be on a higher age when entering pregnancy maternal age associated with increased metabolic and associated changes may act as further imprinting patterns on their children [2, 13, 28, 30]. 

#### 3.2.2. Birth Weights

In a meta-analyses of Harder et al., for example, it could be indicated that higher birth weights (>4000 g) and lower birth weights (<2500 g) tend to be a crucial risk factor for an increased susceptibility for type 2 diabetes [11]. Focusing on the u-shaped curve by following the end of high birth weights, it has been discussed that maternal hyperglycemia, respectively, maternal obesity may lead to increased fetal insulin concentrations causing macrosomia, malformations, and higher birth weights in their offspring [2, 11, 13]. Focusing on the other end of the u-shaped curve by targeting lower birth weights, it has been discussed that prenatal under- or malnutrition could be linked to alterations in the intrauterine environment causing metabolic and cardiovascular diseases postnatal. Nevertheless, low birth weight babies seem to be more affected to obtain overnutrition postnatal causing rapid weight gain leading to obesity and type 2 diabetes in adulthood [2, 8, 11]. 

#### 3.2.3. Maternal Hypercholesterolemia

As already mentioned, maternal hypercholesterolemia could also be connected to early metabolic and cardiovascular changes in their offspring [35, 36]. Napoli et al. suggested that maternal hypercholesterolemia during pregnancy may induce changes in the fetal aorta determining long-term susceptibility of fatty-streak formations as well as typical atherosclerotic lesions [35, 36]. By following this concept, dietary interventions during pregnancy may offer long-term benefits in their offspring [35, 36]. 

## 4. The Uteroplacental Unit

The utero-placental unit displayed an underestimated role within cross-generational programming and gathers more and more attention (Figure 2). Hence, the utero-placental unit reflects a metabolically active system, which seems to be involved, for example, in the transportation of nutrients, oxygen, amino acids, the production of hormones, and the regulation of fetal growth [47–52]. Glucose displays one of the main sources of energy for the human fetuses and the placenta [47–52]. The maternal-fetal glucose consumption is described to be different in pregnancies associated with intrauterine growth retardation (IUGR) [47, 48, 50]. Additionally, amino and fatty acid transports are altered in IUGR fetuses, too [47–52]. Focusing on endocrine functions, the placenta produces several hormones, including estrogens and progesterone, hCG, human GH variant, and human placental lactogen [47–52]. However, several studies have shown associations between concentrations in hormones and embryonic growth velocity, birth weight, and placental weight [47–53]. Additionally, it has been described that an altered utero-placental perfusion and an inadequate trophoblast invasion as well as disturbances in the placental barrier function, for example, glucocorticoid barrier, are linked to IUGR respectively diabetic pregnancies [47–53]. 

Additionally, it has been also suggested that the synthesis as well as secretion of adipocyte-derived hormones, such as leptin, is already regulated due to fetal development [54–57]. Further, it has been described that hypothalamic neuropeptides, which regulate energy intake in adult life, may be already functioning in the fetal brain [54–57]. Furthermore, Plagemann et al. showed in *in vivo *studies an early increase in weight gain and fat depositions followed by hyperphagia, obesity, hyperleptinaemia, hyperglycaemia, and hyperinsulinaemia as well as insulin resistance [54–57]. 

## 5. Molecular Mechanisms of Diabetic and Metabolic Programming

In general, the term “programming” defines perpetuation at a critical period of development causing alterations with lifelong, sometimes irreversible consequences. These multiple mechanisms of programming reflect interplays of genetic and environmental influences at early stages of development modeling diabetic, dysmetabolic, and associated cardiovascular diseases later in life. Looking from the genetic point, the genotype of the fetus can be influenced by mutations. For instance, a mutation in glucokinase or chromosome 6 may result in impaired insulin secretion or in polymorphisms, such as INS-VNTRI polymorphism or polymorphisms in the IGF 1 gene promoter region [58, 59]. Coming from the environmental perspective, mechanisms of influences seem to be more complex and are abrasively divided in adverse intrauterine and adverse postnatal environmental factors. Considering adverse intrauterine environmental parameters, factors such as maternal food restriction, malnutrition, placental dysfunction, obesity, gestational diabetes, hypertension, preeclampsia, and gestational weight gain play an important role. Coming from the adverse postnatal environment perspective, factors like malnutrition, such as high fat diet, early “catch-up” growth [2, 13, 28], inactivity, and aging play an additive role. The current state of knowledge reflects that adverse intrauterine milieu is linked with altered environmental, molecular, and epigenetic changes in offspring of dysmetabolic mothers. 

### 5.1. Pregnancy Acts as a Proinflammatory State—Does Inflammation Play a Key Role?

Yet in early stages of development, placenta cells, such as Hofbauer cells as well as syncytiotrophoblasts and cytotrophoblasts, contribute to local as well as systemic levels of cytokines and inflammatory molecules [60]. In the course of pregnancy, the physiological role of placental produced cytokines is rarely understood. A common hypothesis is that these cytokines contribute to the low grade systemic inflammation during gestation [60]. There is also the assumption that the activation of inflammatory pathways is needed to induce maternal insulin resistance, which is physiological up to a certain grade of insulin resistance during pregnancy. However, in dysmetabolic pregnancies, for instance, complicated by diabetes, continuous adverse stimuli are associated with the dysregulation of metabolic and vascular as well as inflammatory pathways assisted by increased circulating concentration of inflammatory molecules [34, 43, 60]. 

Since pregnancy by itself mimics the Metabolic Syndrome up to a certain level by featuring dysmetabolic conditions, such as obesity as well as insulin resistance, and is frequently accompanied by dyslipidemia and hypertension, even in physiological pregnancies, it is believed that maternal adipose tissue contributes to the inflammatory state of pregnancy, for example, by releasing common molecules as the placenta does [34, 43, 60]. To exemplify, the production of tumor-necrosis-factor- (TNF-) *α* as well as leptin is associated with an increased production for additional inflammatory markers, fibrotic response, vascular remodeling, and protein facilitating lipid storage within the placenta [34, 43, 60]. 

It is described that obesity and type 2 diabetes process stimuli within the adipose cell to increase the production of inflammatory cytokines [34, 43, 60] and that the subsequent recruitment of macrophages enhances the local production of cytokines by adipocytes and stromal cells [34, 43, 60]. Next to cumulative cytokine productions, there are further stimuli, such as oxidative stress and endothelial injury, which are disbalancing metabolic conditions towards dysmetabolic environment [34, 43, 60, 61]. Proinflammatory cytokines, for instance, TNF-*α* and IL-6, affect glucose and lipid metabolism in a negative way and inhibit actions in insulin sensitive tissues [34, 43, 60–64]. However, multiple mechanisms and pathways act simultaneously rectified, as well as opposed by sharing the same or using different pathways. Cytokines may even cause different effects depending on their cell type or developmental stage [34, 43, 60–64].

Even if TNF-*α* and IL-6 bind to different receptor classes, NF*κ*B as a joint transcription factor may be recruited [34, 43, 60–70]. In this course, NF*κ*B regulates several endogenous genes within pro-inflammatory pathways [34, 43, 60–69] and even triggers proinflammatory responses, for example, by binding on the TNF-*α* gene promoters and therefore looping inflammatory response [34, 43, 60–69]. 

Taken together, severe obese as well as diabetic pregnancies are associated with the disbalance of pro-inflammatory pathways supported by increased circulating concentrations of inflammatory molecules [34, 43, 60–69]. In presence, it may assumed that placental pro-inflammatory cytokines, including leptin, which acts as an adipocytokine, as well as adipose tissue contribute to the inflammatory state during pregnancy. It is also described that cytokines have different effects on different organs and developmental stages. To exemplify, an accumulation of TNF-*α* is associated with an increased production of pro-inflammatory markers, fibrotic response, vascular remodeling, and proteins facilitating lipid storage [34, 43, 60–69, 71].

### 5.2. Pregnancy Acts as a State of Oxidative Stress—Does Oxidative Stress Play a Key Role?

Pregnancy is characterized by dynamic changes and increased susceptibility to oxidative stress [15, 72–74]. The susceptibility to oxidative stress reflects results of disturbances within the prooxidant-antioxidant balance due to increasing basal oxygen consumption as well as changes in energy substrate use by multiple organs, including the fetoplacental unity [15, 72–74]. In the early pregnancy, the placenta holds a hypoxic environment. In the course of gestation, it changes to an oxygen-rich environment and its abundant mitochondrial mass promotes the production of reactive oxygen species (ROS) [15, 72–74]. Furthermore, nitric oxide (NO) is also produced by the placenta [15, 72–74] and in addition to other reactive nitrogen species the potential to oxidative stress is increased. Furthermore, the placenta is very rich in macrophages promoting the placental production of free radicals [15, 72–74], For example. 

In a pregnancy with a physiological metabolic state, the placental environment is able to induce protective mechanisms against free radicals as gestation goes on [15, 72–74]. Counterregulation mechanisms in normal pregnancies are modulated by enzymatic inductions and activities such as gluthatione peroxidase, transferase and reductase, or glucose 6-phosphate dehydrogenase [15, 72–78] as well as due to nonenzymatic free radical protectors and scavengers, such as antioxidants like vitamin C and E [15, 72–79]. 

In general, pregnancy is a very sensitive state, in which this equilibrium can be easily disturbed causing dysbalance towards oxidative stress leading to insulin resistance, gestational diabetes, and gestational hypertension, which are often associated [72–78, 80, 81]. Moreover, hyperglycemia per se can promote nonenzymatic glycation, which is able to induce ROS formation in the presence of reactive transitional metals [72–78, 80, 81]. This hypothesis got supported by evidences that elevated ROS in insulin resistance [72–78, 80–82] and diabetes and on the other side the prevention of diabetic fetal malformations by the reduction of oxidative stress seem to be linked [72–78, 80–84]. 

With respect to Simmons et al. understanding of interactions within the pathogenesis of diabetes, mitochondrial dysfunction and oxidative stress got clearly improved [15], so oxidative stress and mitochondrial dysfunction are discussed within pathogenesis of type 2 diabetes as well as in intrauterine growth retardation (IUGR) [2, 15, 85–88]. In fetuses with manifested IUGR, low oxygen levels process the production of free radicals [15, 86–88]. Selak et al. suggested that decreased pyruvate oxidation in muscle mitochondria of IUGR rats is linked to reduced adenosine triphosphate (ATP) production and pyruvate dehydrogenase activity, which may be related to altered ATP synthesis in muscles, e.g., glucose transport, glycogen synthesis causing insulin resistance and type 2 diabetes [87]. Morino et al. suggested that “defects in mitochondrial oxidative phosphorylation activity in […] offspring of parents with type 2 diabetes” may be linked [88]. There is rich evidence that embryos, fetuses, and children of diabetic mothers show higher rates of malformations and anomalies, which seems to be related to mechanisms of oxidative stress, hyperglycemia-induced ROS, mitochondrial dysfunction and altered glycosylation, peroxidation, and so forth [15, 72, 89–93]. 

Little is known about vulnerable time windows during pregnancy, in which dysbalances towards oxidative stress triggering insulin resistance and diabetes might happen. There is evidence that activation of the hexosamine pathway causes oxidative stress through depletion of GSH altering gene expression, which may lead to malformations and anomalies [91]. Other investigations describe a correlation between maternal and fetal oxidative stress by analyzing umbilical cord blood and infant's blood, for example, up to an age of 3 days [93]. However, there are reports that antioxidants improve early fetal damage [72]. Furthermore, it could be displayed by analyzing *in vivo* studies that the supplementation of antioxidants, such as Vitamin E, may reduce the rates of malformations [84]. However, as the case may be further analyses have to be done.

## 6. Epigenetic Mechanisms of Diabetic and Metabolic Programming

Epigenetic mechanisms are clearly important pathways altering the developmental genome. Hence, epigenetic conditions can be shifted by different environmental factors leading to an adverse intrauterine, but also to an adverse postnatal environment [94, 95]. With great respect to Rebecca Simmons and her work the next section will focus basically on her expertise. In our opinion she already discribed mechanisms of epigenetic regulations particularly [95]. By citing Simmons an outstanding characterization is given. “An adverse intrauterine milieu can impact the development of the fetus by modifying gene expression in both pluripotential cells and terminally differentiated, poorly replicating cells. The long-range effects on the offspring (into adulthood) depend upon cells undergoing differentiation, proliferation, or functional maturation at the time of the disturbance in maternal fuel economy” [95]. Currently it is known that “there are at least two distinct classes of epigenetic information that can be inherited with chromosomes” [95]. 

One “class of epigenetic regulation is DNA methylation, in which a nucleic acid base is modified by a DNA methyltransferase at the C5 position of cytosine” [95]. “DNA methylation is […] associated with gene silencing and contributes to X-chromosomal inactivation, genomic imprinting as well as transcriptional regulation of tissue-specific genes during cellular differentiation” [95]. 

The other “class of epigenetic control of gene expression involves changes in chromatin proteins” causing histone modifications [95] “by acetylation, methylation, sumoylation, phosphorylation, glycosylation, and ADP ribosylation” [95]. In general, “the most common modifications involve acetylation and methylation of lysine residues” of the histones H3 and H4 [95]. “Increased acetylation induces transcription activation whereas decreased acetylation usually induces transcription repression” [95].

*In vivo* studies were able to show that “abnormal intrauterine environment induces epigenetic modifications of key genes,” which are, for instance, involved in the regulation of *β*-cell development or causing “hypomethylation and hyperacetylation of genomic DNA in brain and liver” [95], for example. “Hypomethylation of the glucocorticoid receptor” […] “in the liver of the offspring” can also be induced by maternal low protein intake [95]. Within this context, important experiments were done by using agouti mice [95, 96]. “In the agouti mouse, mutations in the regulatory region of the agouti locus cause mice bearing the dominant “viable yellow” (Avy), "IAPyellow" (Aiapy), or "hypervariable yellow" (Ahvy) alleles to synthesize […] more pheomelanin than eumelanin” [95]. However, modifications “by methylation reside within parasitic DNA elements or retrotransposons, such as endogenous retrovirus” [95]. Under normal circumstances “a cryptic promoter within the retrotransposon is silenced by methylation allowing normal tissue-specific and regulated agouti expression” [95]. Under hypomethylation, the promoter gets active and “drives constitutive ectopic expression of the agouti gene, leading to […] obesity, hyperinsulinemia, diabetes, increased somatic growth, and increased susceptibility to hyperplasia, and tumorigenesis” [95]. Moreover, it could be “shown that the methylation status of these inserted viral DNA sequences can be modified by the methionine, folic acid and choline content of the maternal diet” [95]. By adding “methyl donors to the maternal diet […] methylation of the retrotransposon” was increased leading to suppressed ectopic gene expression and improved outcome in the offspring [95–98]. But “epigenetic modification of gene expression” can also happen in the postnatal period [95]. Weaver et al. showed that “stress-induced behaviors of rat mothers during lactation” period induced DNA methylation in their suckling offspring [95].

## 7. Conclusions

Facing an obese and diabetic epidemic, which affects all generations, especially younger generations, and experiencing already that obese as well as diabetic mothers increase the risk of obesity and type 2 diabetes in their offspring significantly at an early stage of life by “programming” them metabolically, but also for cardiovascular disease later in life, the research on cross-generational programming has a fundamental impact in understanding novel molecular mechanisms of imprinting diabetes, obesity, and cardiovascular disease. 

In conclusion, it could be shown that different mechanisms are able to alter the intrauterine environment. However, cross-generational programming of metabolic and cardiovascular diseases is still poorly understood and further investigations will give novel insights targeting these the complex imprinting mechanisms.
